# Hypnotism during Parturition

**Published:** 1886-02

**Authors:** 


### Hypnotism During Parturition.

In the *Wiener Medizinische Wochenschrift* (No. 45), Dr. E. Pritzel, assistant to Prof. Karl Braun, reports a case of childbirth during hypnotism, probably the first of the kind on record.

The patient, single, 26 years of age, was admitted to the obstetrical wards of Prof. Braun in the eighth month of pregnancy. She had always menstruated regularly from her fourteenth year up to January of last year. Since then her health had been good, and she had been entirely free from the nervous manifestations so common during pregnancy.

Upon examination, it was found that she was readily thrown into a hypnotic state by holding a thermometer bulb before her eyes for a few moments. She became unconscious and insensible to all irritations, while her color, pulse and pupillary reactions remained unaffected. At the end of a quarter or half an hour, the patient was aroused by long and continued irritation, shaking the body, blowing upon the cornea, slapping the breast with cold, wet cloths, etc. After revival, she declared that she felt well after each experiment, but it was noticed that it was usually followed by a deep, but natural, sleep.

Labor set in October 30th, and during the first stage she was restless and unmanageable. Cramps in the limbs and intensity of the pains during the second stage suggested a narcotic or the induction of artificial hypnotism. The latter expedient was adopted, and with entire success. She became unconscious and i nsensible. It was found that, instead of decreasing in force, the uterine contractions became more energetic, and were aided by the abdominal muscles. While the patient was entirely insensible, it was still noticed that she bent the left forearm as if cramp were present, and there was considerable stiffness in the left leg. The right side of the body was unaffected. Between the pains she lay motionless, as if asleep. A well-developed female child was born, which cried lustily; the placenta was expelled, under the influence of abdominal pressure, in three-quarters of an hour. The patient was wakened by holding ammonia to her nose and shaking her, after unconsciousness lasting an hour and a quarter. The confinement was in every other respect strictly physiological. In two other cases from the same clinic the method was also successful, although to a less marked degree.—*Medical Times.*

